# Information, Entropy, Life, and the Universe

**DOI:** 10.3390/e24111636

**Published:** 2022-11-10

**Authors:** Arieh Ben-Naim

**Affiliations:** Department of Physical Chemistry, The Hebrew University of Jerusalem, Jerusalem 91904, Israel; ariehbennaim@gmail.com

**Keywords:** information, Shannon’s measure of information, entropy, thermodynamics, the second law, life and the universe

## Abstract

In 2015, I wrote a book with the same title as this article. The book’s subtitle is: “*What we know and what we do not know.*” On the book’s dedication page, I wrote: **“*This book is dedicated to readers of popular science books who are baffled, perplexed, puzzled, astonished, confused, and discombobulated by reading about Information, Entropy, Life and the Universe*.”** In the first part of this article, I will present the definitions of two central concepts: the “Shannon measure of information” (SMI), in Information Theory, and “Entropy”, in Thermodynamics. Following these definitions, I will discuss the *framework of their applicability.* In the second part of the article, I will examine the question of whether *living systems* and the *entire universe* are, or are not within the *framework of applicability* of the concepts of SMI and Entropy. I will show that much of the confusion that exists in the literature arises because of people’s ignorance about the *framework of applicability* of these concepts.

## 1. Introduction

**In**Section 2, we discuss the general concept of “information”, whose meaning everyone seems to know, but whose definition is elusive. Next, we briefly discuss the so-called Shannon’s “Information theory” and its central concept, “Shannon’s measure of information” (SMI). We discuss Shannon’s motivation for defining the SMI and some interpretations of the SMI. Finally, we introduce the unit of information: the *bit.*

In Section 3, we discuss Entropy, the central concept of thermodynamics. We briefly introduce three definitions of entropy, which, although quite different from each other, are equivalent in some sense. We also show that the *framework of applicability* of this concept is already embedded in its definition. It is unfortunate that so many authors who write about entropy and the Second Law either forget or ignore the *framework of applicability* of these concepts. It is appropriate here to quote Einstein’s statement on thermodynamics:

“***[Thermodynamics], is the only physical theory of universal content, which I am convinced, that within the framework of applicability of its basic concepts will never be overthrown.***”

In Section 4 and Section 5, we examine the main question posed in this article as well as in this Special Issue of Entropy. The SMI and Entropy are well-defined concepts. They were, and are still applied successfully to specific and well-defined aspects of life and of the universe, for example, specific chemical reactions in biology, heat engines and phase transitions in thermodynamics, and many more. The question that begs to be answered is whether these two concepts are applicable or not to either an *entire living system* or to the *entire universe*. I will demonstrate that neither an *entire living system* nor the *entire universe* falls within the framework of applicability of either the SMI or Entropy. This is the major reason that has caused so much confusion in both Information theory and in thermodynamics.

## 2. Information and Information Theory

This section tackles the distinction between two concepts: first, the general concept of *information*, and second, a *specific* kind of information for which a *specific measure* was developed by Shannon [1]. More specifically, we shall introduce the *Shannon measure* of *information* (SMI), providing its definition, its various interpretations, and a few of its applications.

Information is a very general concept. We all know what information means, yet its formal definition eludes us. We know that Information can either be true or false, and it can be important or irrelevant. Information might be redundant, ambiguous, or meaningless, or it can be objective or highly subjective. On top of these, some information can be measured. We will discuss below a *specific measure* of information that was introduced by Shannon, in 1948.

On one hand, two messages might have different “sizes”, but they convey the same information. On the other hand, two messages might have the same “size”, but they convey very different information.

For example, “*Jacob loves Rachel*” and “*Rachel loves Jacob*” have the same size, but they convey different information.

Another example is: “The population of this village is two thousand six hundred and fifty-five”, and “The population of this village is 2655.

Obviously, these two pieces of information convey the same message, but have different sizes.

Below, we shall introduce Shannon’s measure of information (SMI), which depends not only on the number of letters but also on the frequencies of the various letters in a particular language. In 1948, Shannon published a remarkable article [1] entitled:

**“A Mathematical Theory of Communication.”**

In Section 6 of this paper, Shannon describes the measure he discovered:

“*Suppose we have a set of possible events whose probabilities of occurrence are, $p_{1},p_{2},\cdots ,p_{n}$. These probabilities are known but that is all we know concerning which event will occur. Can we find a measure of how much “choice” is involved in the selection of the event or how uncertain we are of the outcome?*”

“*If there is such a measure, say $\left(p_{1},p_{2},\cdots ,p_{n}\right)$, it is reasonable to require of it the following properties:*”

1. H should be continuous in the $p_{i}$.
2. If all the $p_{i}$ are equal, $p_{i}=\frac{1}{n}$, then H should be a monotonic increasing function of n. With equally likely events, there is more choice, or there is uncertainty when there are more possible events.
3. If a choice be broken down into two successive choices, the original H should be the weighted sum of the individual values of H.

Then, Shannon proved that:

“*The only H satisfying the three assumptions above is the form:*”

(1) $$H=-K\sum p_{i}\log p_{i}$$

In this article, we are only interested in the interpretation of the SMI, and its *meaning* as a measure of information. The relevance of H to thermodynamics will be discussed in Section 3. Most of Shannon’s article is concerned with the Theory of *Communication* (including problems of coding and decoding, the efficiency of transmission of information, etc.). 

It should be emphasized that Shannon formulated his problem in terms of a *probability distribution*, $p_{1},\cdots p_{n}$. He sought a measure of how much “choice” or “uncertainty” there is in the outcome of an experiment. Later, he referred to the quantity *H*, defined in Equation (1), as a measure of “information, choice and uncertainty”. Clearly, Shannon did not seek a measure of the *general concept of information*, but only a *measure* of *information contained in* or *associated with* a probability distribution. This is a very important point that one should remember but which has unfortunately been ignored or forgotten by many authors of popular science books. More details are available in references [2,3,4,5]. Shannon was not interested in thermodynamics in general, nor in *entropy* in particular. However, he noted that “*the form of H will be recognized as that of entropy as defined in certain formulations of statistical mechanics*…” Therefore, he called the quantity *H* “*the entropy of the set of probabilities* $p_{1},\cdots p_{n}$”. 

As we shall see in Section 3, entropy is a special case of SMI; it is defined only on tiny small sets of probability distributions. Thus, only when *H* is applied to those distributions used in statistical mechanics does it become identical to the statistical mechanical entropy. 

In my opinion, naming the SMI “entropy” was an unfortunate mistake. This has caused a great deal of confusion in Information Theory and in thermodynamics. It should be noted here that many authors confuse Entropy (the thermodynamic entropy) with SMI (referred to as Shannon entropy), and SMI with Information. A typical example may be found in Gleick (2011) [6]:

“*Indeed, H is ubiquitous, conventionally called entropy of a message, or Shannon entropy, or simply, the information*.”

Let us discuss briefly the meaning of SMI. There are essentially three interpretations of the SMI. The first is an *average of the uncertainty* about the outcome of an experiment, the second is an *average measure of the unlikelihood*, and the third is a measure of information. 

The interpretation of *H* as an *average uncertainty* is quite common in the literature. This interpretation follows directly from the meaning of the probability distribution. Briefly, we are given an experiment having *n* possible outcomes with probability distribution $p_{1},\ldots ,p_{n}$. The larger the value of $-\log\;p_{i}$, the larger the extent of uncertainty about the occurrence of the event *i*. Therefore, multiplying $-\log\;p_{i}$ by $p_{i}$, and summing over the index *i*, we obtain an *average* uncertainty about all the possible outcomes of the experiment. 

A slightly different but still useful interpretation of *H* is in terms of *likelihood* or *expectedness*. This interpretation is also derived from the meaning of probability. When $p_{i}$ is small, event *i* is unlikely to occur, or its occurrence is less expected. Therefore, the larger the likelihood or the larger the expectedness for the event since $0\leq p_{i}\leq 1$, it follows that $-\infty \leq \log\;p_{i}\leq 0$. The quantity $-\log\;p_{i}$ is thus a measure of the *unlikelihood* or the *unexpectedness* of the event *i*. Therefore, the quantity $H=-\sum p_{i}\log\;p_{i}$ is a measure of the *average unlikelihood* of the entire set of events.

Ironically, the interpretation of *H* as a “measure of information” is not straightforward. It does not follow the definition of *H.* It is also not a measure of any type of information, but rather of a very particular kind of information. 

As in the case of the other two interpretations of the SMI, some authors assign to the quantity $-\log\;p_{i}$ the meaning of information (or self-information) associated with the event *i*, and claim that the SMI is a measure of the “average” information. Many authors explain that if an event is rare, i.e., $p_{i}$ is small hence, hence $-\log\;p_{i}$ is large, hence one gets “more information” when that particular event has occurred. This interpretation of probability is invalid. When I am informed that an event *i* has occurred, I obtain the *information* on the occurrence of the event *i*. I might be surprised to learn that a rare event has occurred, but the size of the *information* I obtained when the event occurred was not related to the probability of that event.

The best way of interpreting *H* as a measure of information is by using the 20-question game. This is discussed lengthily in Ben-Naim (2017) [7]. Here, we just say that the SMI is a measure of the “amount of information” one needs to obtain by asking binary questions in order to find out which outcome has occurred given the probability distributions of all possible outcomes of that game. It is also equal to the *average* number of questions you need to ask in order to obtain this information. 

To conclude, the interpretation of the SMI as *information* should be made with extreme caution. First, because SMI is not *information*, and second, because it is a very specific measure of a very specific kind of information. 

Thus, the SMI does not measure *the average amount of information* contained in the message. It is in some sense only a measure of the “size” of the message. It may be *interpreted* as a “measure of a message”, but that measure has nothing to do with the information carried by the message. It is related to the distribution of letters in an alphabet of a specific language. 

Another common misconception is about bits and information. Bits are *units* of information. However, one cannot use bits as a measure of *any* information simply because not every piece of information is measurable. 

We next discuss the units of information by using an example of an experiment having only two outcomes. This example will also clarify the meaning of the quantity *H* as a measure of information.

Consider the case of an experiment, or a game with only two outcomes, N=2. Suppose that a coin is hidden in one of two boxes, and you have to find out the coin’s location by asking binary questions. You also know the probability distribution, i.e., you know the probability of finding the coin in each of the two boxes.

Intuitively, we feel that if the probability distribution is not symmetric for the two boxes, we have more *information* than when the probability distribution is symmetric. To clarify this point, consider the following three cases:

(a) Symmetric distribution: (½, ½)
(b) Asymmetric distribution: (p,1−p), (0<p< ½)
(c) Extreme asymmetric distribution: (0,1)

In the first case (a), you must ask one question in order to know where the coin is. In the third case (c), you do not have to ask any questions; given the distribution (0,1) is equivalent to having the knowledge that the coin is in the second box, i.e., you *know* where the coin is. In any intermediate case (b) of $1<p<\frac{1}{2}$, you feel that you “know” more than in the first case (a), but less than in the third case (c). On the other hand, in all cases except the third one, one must ask *one* question in order to find out where the coin is. Clearly, we cannot use the equation log 2 = 1 to quantify the amount of “information”, we have in the general case of (p,1−p). To do so, we modify the rules of the game or the task we are facing.

Again, suppose that you know that the coin is in one of the boxes, and you are given the distribution (p,1−p). You choose one box and ask, “Is the coin in that box?” If the answer is YES, you win, but if the answer is NO, you must continue and ask, “Is the coin in the second box?” The answer you will certainly obtain is, YES.

The difference between the rules of this game and the previous ones is simple. When you have only two possibilities, you will *know* where the coin is after the first question, whatever the distribution is, and whatever the question is. However, if the answer to the first question in the modified game is NO, we need one more *verifying* question in order to obtain a YES answer. In other words, the game ends when you obtain a YES answer to your question. Clearly, in this modified game you will not need to ask any questions in case (c), and you will need on average to ask more questions in (a) than in case (b). 

We define the SMI for this game by:

(2) $$H\left(p\right)=-p\;\log_{2}p-\left(1-p\right)\log_{2}\left(1-p\right)$$

**Figure 1:**
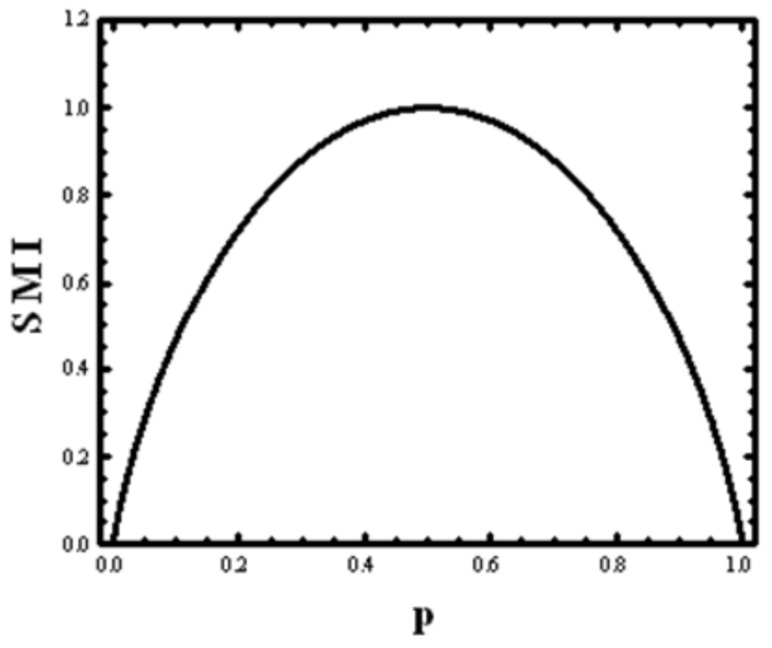
The function *H(p)* for the case of two outcomes, Equation (2).

This function is shown in Figure 1. It has a maximum at $p=\frac{1}{2}$, and a minimum at both p=0 and p=1. If we choose the base 2 for the logarithm, then the maximal value of *H* (*p*) is:

$$H\left(p=\frac{1}{2}\right)=-\frac{1}{2}\log_{2}\frac{1}{2}-\frac{1}{2}\log_{2}\frac{1}{2}=1$$

This value is used as the *unit* of *information* called *bit* (short for *b*inary digi*t*). Note carefully that this unit of information measures the amount of information one obtains from asking a question answerable with a YES or NO, when the probabilities of the two outcomes are equal. If the probabilities are not equal, the value of the SMI can have any value between zero and one. 

In some recent popular books one finds statements claiming that a *bit* is the *atom* of the information. It is not! An atom is the “atom” of a chemical element. A single molecule is the “atom” of a compound, and perhaps one can say that a “gene” is the “atom” of heredity, but the “bit” is a *unit* of information like a centimeter is a unit of length, and gram is a unit of mass. 

In the introduction of Lloyd’s book (2006) [8], we find:

“*This is the story of the bit and the universe. The universe is the biggest thing there is and the bit is the smallest possible chunk of information. The universe is made of bits. Every molecule, atom and elementary particle registered bits of information*.”

Clearly, by definition, the universe is the *biggest* thing. However, the “bit” is a *unit* of information, not *information*, and certainly not the “*smallest possible chunk of information.*” 

The claim that the “universe is made of bits” is quite common. It follows from the famous “It from bit” idea of Wheeler (1990) [9], see below. Suffice it to say that the “bit” is the *largest* amount of information one can obtain from a question answerable with YES/NO. The *smallest* amount of information one can obtain from such a question is *zero*!

Thus, for any binary question, the answer provides the amount of information between zero to one bit, calculated by the corresponding SMI.

Recently, one can read in some popular science books numerous statements about *information* being *physical*, that it obeys some physical laws, and that everything is *information*. This came about as some have subscribed to the most famous statement made by Wheeler: “It from bit”, which conveys the idea that all physical things are informational-theoretical in origin.

This is the most misleading “slogan”. If you kick a stone, what will most likely happen is that it will either fly away or you will hurt your foot. You cannot kick the information on my date of birth or the information given in this article. A bit is a unit of information, it is not a physical entity!

Here, is another quotation regarding information as being “physical”, Wheeler (1990) [9]:

“*It from bit. Otherwise put, every “it”–every particle, every field of force, even the space-time continuum itself–derives its function, its meaning, its very existence entirely–even if in some contexts indirectly–from the apparatus-elicited answers to yes-or-no questions, binary choices, bits. “It from bit” symbolizes the idea that every item of the physical world has at bottom–a very deep bottom, in most instances–an immaterial source and explanation; that which we call reality arises in the last analysis from the posing of yes-or-no questions and the registering of equipment-evoked responses; in short, that all things physical, are information-theoretic in origin and that this is a participatory universe*.”

These kinds of statements have misled numerous scientists, as well as popular-science writers, and have unfortunately led practitioners in the field of science to claim that “information theory” is a revolution in *physics*, in a similar way to or perhaps greater than the two great revolutions in physics: relativity and quantum mechanics. I personally maintain that information theory is not a theory of information (but a theory of communication); it is certainly not a theory of physics. Therefore, the creation of “information theory” was not, and is not a revolution in physics.

Wheeler’s slogan “it from bit” embodies the widespread confusion between the three concepts: Information, SMI and the bit. Based on what Wheeler wrote, it is clear that what he meant to say is that “Everything is Information”. I certainly do not share the same view. However, what Wheeler actually says is quite different from what he intended to say. Even if one accepts the view that every physical object is information, one cannot say that every physical object is SMI, and certainly not a bit!

Seife (2007) [10] made the following statement.

“*Information theory is so powerful because information is physical. Information is not an abstract concept, and it is not just facts or figures, dates, and names. It is a concrete property of matter and energy that is quantifiable and measurable,” … and “And everything in the universe must obey the laws of information because everything in the universe is shaped by the information it contains*.”

Such statements have fanned the flames of confusion between “information”, the “measure of information” and “physical objects.” Information *is* an abstract concept, but it *is not* a concrete property of matter and energy. The cup of tea you are holding in your hand was not “shaped by the information it contains” (whatever the verb “shaped” means).

In concluding this section, we stress again that one must make the distinction between the general colloquial term *information* on one hand, and the specific measure of information as used in Information Theory, on the other hand. The central quantity in Information Theory is the Shannon measure of information (SMI). It is quite important to acknowledge that the SMI is a specific measure of the size of the message that carries the information, and is not concerned with the meaning, value, importance, etc. of the information itself.

Confusing the colloquial information with the SMI is commonplace in the literature. This confusion is prevalent especially when *information* is used in connection with *entropy*, life, and the universe. We shall discuss a few examples in the following sections. 

## 3. Three Different but Equivalent Definitions of Entropy

In this section, we start with the presentation of three different but equivalent definitions of entropy. By “different”, we mean that one definition does not follow the other. By “equivalent”, we mean that for any process in which the change in entropy can be calculated, we obtain the same results by using any one of the three definitions. Clearly, the equivalency of the three definitions does not imply identity. To the best of my knowledge, no one has ever shown that either the Boltzmann or the ABN (see below) definition can be derived from Clausius’. In fact, since the three different definitions are based on totally different and unrelated concepts it is almost a miracle that these three definitions are equivalent in the sense stated above.

The third definition, which I refer to as the Ben-Naim (ABN) definition is based on Shannon’s measure of information (SMI) [1,7]. This definition of entropy has some advantages. It is the only definition that provides a simple, intuitive, and meaningful interpretation of entropy and the Second Law. It should be noted that nowadays, people refer to SMI as entropy. When I refer to the ABN definition of entropy, I mean the thermodynamic entropy, derived from SMI. The ABN definition also provides directly the *entropy function* of an ideal gas and, by extension, also the entropy function for a system of interacting particles (by “entropy function”, I mean an explicit formula for calculating entropy for a well-defined system).

**Clausius’ “definition” of entropy**

**Figure 2:**
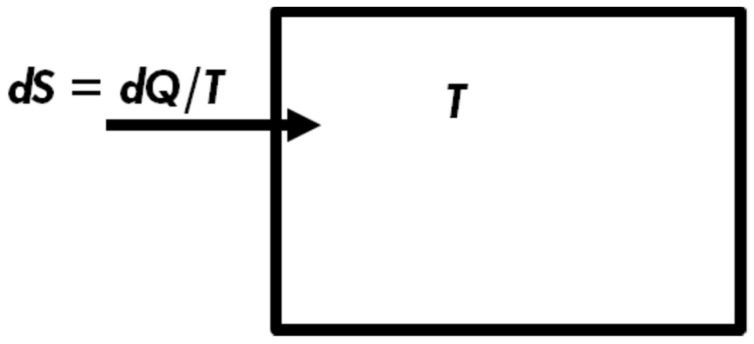
Clausius’ definition of a small change in entropy dS, for the process of transferring a small amount of heat *dQ* to a system at a constant temperature *T*.

First, note that I enclosed the word “definition” in inverted commas. The reason is that Clausius did not really *define* entropy. Instead, he defined a small change in entropy for one very *specific* process. Clausius started from one particular process; the spontaneous flow of heat from a hot to a cold body, Figure 2. Based on this specific process, Clausius defined a new quantity which he called Entropy. Let dQ>0 be a *small quantity* of heat flowing *into* a system, being at a constant temperature *T*. The *change* in *entropy* was defined as:

(3) $$dS=\frac{dQ}{T}$$

The letter *d* does not mean a differential quantity, but rather for a *very small quantity*, *T* is the absolute temperature, and *Q* has the units of *energy*. Therefore, the entropy change has the units of *energy* divided by units of *temperature*. Some authors add the subscript “rev” in the Clausius definition, which means that Equation (3) is valid only for a “reversible” process. This is unnecessary. In fact, the additional requirement that the process is “reversible” may even be confusing; the term “reversible” (or irreversible) has many different meanings. See Ben-Naim (2017, 2020) [5,11]. 

The quantity of heat dQ *must* be very small, such that when it is transferred into, or out from the system, the temperature *T* does not change. If dQ is a finite quantity of heat, and one transfers it to a system which is initially at a given *T*, the temperature of the system might change, and therefore the change in entropy will depend on both the initial and the final temperature of the system. Note carefully that this equation does not define *entropy* but only changes in entropy for a particular process, i.e., a *small* exchange of heat (dQ>0 means heat flows into the system, dQ<0 means heat flows out of the system. Correspondingly, dS will be positive or negative when heat flows into, or out from the system, respectively).

Clausius’ definition in thermodynamics is used for calculating entropy changes even for processes that do not involve heat transfer. For examples, see Ben-Naim, references [4,5,11]. This could not have been possible without the recognition (originally postulated) that entropy is a *state function*. Once we accept that entropy is a state function we could devise a *path* leading from an initial state to a final state for which the entropy change can be calculated. As an example, consider the expansion of an ideal gas from volume *V* to volume 2*V*. If this process is carried out in an isolated system, there is no heat transfer involved, yet there is a change in entropy that can be calculated. For details, see references [4,5,11,12]. The result for one mole of ideal gas is:

(4) ΔS=Rln2

In Equation (4), *R* is the gas constant: 8.3144 J⋅mol^−1^⋅K^−1^ = 1.987 cal⋅mol^−1^⋅K^−1^

It is not uncommon to refer to the equation dS=dQ/T as Clausius’ *definition* of entropy. However, this equation does not define entropy, nor *changes* in entropy for every process (e.g., expansion of an ideal gas). It should be added that even before a proper definition of entropy was posited, it was realized that entropy is a *state function* which means that whenever the *macroscopic state* of a system is specified, its entropy is determined. A well-defined macroscopic state must be an equilibrium state. 

Clausius’ definition, together with the Third Law of Thermodynamics, led to the calculation of “absolute values” of the entropy of many substances. It is ironic to mention that Clausius, the person who coined the term “entropy”, committed the first and perhaps the most egregious error in using the term entropy far beyond the “framework of its applicability”. We shall discuss this in Section 5. 

To summarize, Clausius’ definition in Equation (3) requires the system to be at *thermal equilibrium*. However, in order to calculate finite changes in entropy or absolute entropies based on the third law, one must apply the concept of entropy to systems at equilibrium.

**Boltzmann’s definition**

Boltzmann defined entropy in terms of the *total number of accessible micro-states* of a system consisting of a huge number of particles characterized by the macroscopic parameters of energy *E*, volume *V,* and number of particles *N*.

What are these “number of micro-states”, and how are they related to entropy?

In “classical” systems, each simple particle’s micro-state may be described by its location vector $R_{i}$ and its velocity vector $v_{i}$. Simple particles are those with no internal degrees of freedom. Atoms such as argon, neon, and the like are considered as simple. They all have internal degrees of freedom, but these are assumed to be unchanged in all the processes as discussed in thermodynamics. If we have a system containing *N* simple particles, the configuration or the *micro-state* of the system requires the specification of all the locations and all the velocities (or momenta) of the particles consisting of a *micro-state* of the system. In quantum mechanics, one usually defines *W* as the total number of quantum mechanical solutions of the Schrödinger equation for a system described by its energy *E,* its volume *V* and the total number of particles *N.* We use the shorthand notation $\left(E,V,N\right)$ to describe the macro-state of an isolated system. 

Boltzmann postulated the relationship, which is now known as the Boltzmann entropy:

(5) $$S=k_{B}\log W$$

where $k_{B}$ is a constant now known as the Boltzmann constant (1.380 × 10^−23^ J/K), and *W* is the number of accessible micro-states of the system. The “log” is the natural logarithm. At first glance, Boltzmann’s entropy seems to be completely different and unrelated to Clausius’ entropy. Nevertheless, in all cases in which one can calculate changes in entropy, one obtains agreements between the values calculated by the two methods. 

Although there is no formal proof that Boltzmann’s entropy is equal to the thermodynamic entropy as defined by Clausius, it is widely believed that this is true.

Boltzmann’s entropy as defined in Equation (5) has raised confusion as to whether entropy is, or is not a subjective quantity. One example of this confusion that features in many popular science books is the following: As we shall see below, entropy is related to the “amount of information” about the micro-states of the system. If we “know” that the system is at some specific micro-state, then the entropy is zero. Since “information” is confused with the SMI, and since information might be subjective, some concluded that entropy is also a subjective quantity.

Confusion also stems from misunderstanding *W*, which is the *total* number of accessible micro-states of the system. If W=1, then the entropy of the system is indeed zero (as it is for many substances at 0 K). However, if there are *W* states and we know the specific micro-state in which the system is, the entropy is still k ln W and not zero! This argument led some authors to reject the “informational interpretation” of entropy. In the definition of entropy, *W* includes *all possible micro-states.*

Although it was not explicitly stated, Boltzmann’s definition of entropy applies to an equilibrium state. In statistical mechanics, the Boltzmann entropy applies to the so-called micro-canonical ensemble, i.e., to systems having a fixed energy *E*, volume *V*, and number of particles *N* (*N* could be a vector comprising the number of particles of each species $(N_{1},\ldots ,N_{c}$).

In general, the Boltzmann entropy does not provide an explicit entropy function. However, one can derive an entropy function based on Boltzmann’s definition for some specific systems. The most famous case is an ideal gas, for which one can derive an explicit *entropy function*. This function was derived by Sackur and by Tetrode in 1912 using Boltzmann’s definition of entropy. 

For an ideal gas consisting of *N* simple particles in volume *V*, with a total energy *E*, the total number of micro-state was calculated. The entropy was calculated based on Equation (5). This entropy function is:

(6) SE,V,N=NkBlnVNEN32+32kBN53+ln4πm3h2

We shall see below that this equation may be re-derived based on Shannon’s measure of information.

**Ben-Naim’s (ABN) definition of entropy based on Shannon’s Measure of Information**

This is a relatively recent definition of entropy. It was originally used as an interpretation of entropy, Ben-Naim, 2008 [13,14], but later turned into a definition of entropy referred to as ABN’s definition. This definition is superior to both the Clausius and the Boltzmann definitions. Unlike the Clausius definition, which provides only a definition of changes in entropy, the present one provides the *entropy function* itself. Unlike Boltzmann’s definition, which is strictly valid for isolated systems and does not provide a simple intuitive interpretation, the present one is more general and provides a clear, simple, and intuitive “informational” interpretation of entropy. This definition of entropy also removes any trace of mystery associated with entropy.

In this section, we shall not derive the ABN definition as it is quite long and requires some knowledge of Information Theory. We shall instead outline the steps leading from the Shannon measure of information (SMI) to the thermodynamic entropy function as shown in Equation (6). For more details, see references [7,11]. 

We start with the definition of the SMI, for any given distribution $p_{1},\ldots ,p_{n}$ by:

(7) $$H\left(p_{1},\ldots ,p_{n}\right)=-\sum_{i=1}^{n}p_{i}\log p_{i}$$

The logarithm is to the base 2. The derivation consists of four steps:

**First step: The locational SMI of a particle in a 1D box of length *L***

Suppose we have a particle confined to a one-dimensional (1D) “box” of length *L*. Since there are infinite points in which the particle can be within the interval (0, *L*), the corresponding locational SMI must be infinite. However, we can define as Shannon did the following quantity by analogy with the discrete case:

(8) $$H\left(X\right)=-\int f\left(x\right)\log f\left(x\right)dx$$

This quantity might either converge or diverge, but in practice, we shall use only differences between such quantities. It is easy to calculate the density distribution which maximizes the locational SMI, $H\left(X\right)$ in (8). The result is:

(9) $$f_{eq}\left(x\right)=\frac{1}{L}$$

This is a uniform distribution. The use of the subscript *eq.* (for equilibrium) will be clarified later. The corresponding SMI calculated by substituting (9) in (8) is:

(10) $$H\left(\mathrm{locations}\;\mathrm{in}\;1D\right)=\log L$$

**Second step: The velocity SMI of a particle in a 1D “box” of length *L***

The mathematical problem is to calculate the probability distribution that maximizes the continuous SMI which is subject to two conditions, a normalization condition, and a constant variation. The result is the Normal distribution:

(11) $$f_{eq}\left(x\right)=\frac{\exp\left[-x^{2}/2\sigma^{2}\right]}{\sqrt{2\pi \sigma^{2}}}$$

The subscript eq, which stands for equilibrium, will be clarified once we see that this is the equilibrium distribution of velocities. 

Similarly, we can write the momentum distribution in 1D, by transforming from $v_{x}\to p_{x}=mv_{x}$, to obtain:

(12) $$f_{eq}\left(p_{x}\right)=\frac{1}{\sqrt{2\pi mk_{B}T}}\;\exp\left[\frac{-p_{x}^{2}}{2mk_{B}T}\right]$$

The corresponding maximum SMI is:

(13) $$H_{max}\left(momentum\;in\;1D\right)=\frac{1}{2}\log\left(2\pi emk_{B}T\right)$$

**Third step: Combining the SMI for the location and momentum of one particle; introducing the uncertainty principle.**

In the previous two subsections, we derived the expressions for the locational and the momentum SMI of one particle in a 1D system. We now combine the two results. Assuming that the location and the momentum (or velocity) of the particles are independent events we write:

(14) $$H_{max}\left(location\;and\;momentum\right)=H_{max}\left(location\right)+H_{max}\left(momentum\right)$$

However, quantum mechanics impose restrictions on the accuracy in determining both the location *x* and the corresponding momentum $p_{x}$. We must acknowledge that nature imposes on us a limit on the accuracy in which we can determine *simultaneously* the location and the corresponding momentum. Introducing this uncertainty principle, instead of Equation (14), we obtain the result:

(15) $$H_{max}\left(location\;and\;momentum\right)=\log\left[\frac{L\sqrt{2\pi emk_{B}T}}{h}\right]$$

where *h* is the Planck constant, $h=6.626\times 10^{-34}\;J\;s$.

We next consider one simple particle in a cubic box of volume *V*. We assume that the location of the particle along the three axes *x*, *y,* and *z* are independent. Therefore, we can write the SMI of the location of the particle in a cube of edges *L*, and volume *V* as:

(16) $$H\left(location\;in\;3D\right)=3H_{max}\left(location\;in\;1D\right)=3\log\;L=\log\;V\;$$

Similarly, for the momentum of the particle we assume that the momentum (or the velocity) along the three axes *x*, *y,* and *z* are independent. Hence, we write:

(17) $$H_{max}\left(momentum\;in\;3D\right)=3H_{max}\left(momentum\;in\;1D\right)$$

We combine the SMI of the locations and momenta of one particle in a box of volume *V = L^3^*, taking into account the uncertainty principle. The result is:

(18) $$H_{max}\left(location\;and\;momentum\;in\;3D\right)=3\log\left[\frac{L\sqrt{2\pi emk_{B}T}}{h}\right]$$

**Step four: The SMI of locations and momenta of *N* independent and indistinguishable particles in a box of volume *V***

The next and final step is to proceed from one particle in a box to *N* independent particles in a box of volume *V*. Given the location $\left(x,y,z\right)$ and the momentum $\left(p_{x},p_{y},p_{z}\right)$ of one particle within the box we say that we know the *micro-state* of the particle. If there are *N* particles in the box and if their micro-states are independent, we can write the SMI of *N* such particles simply as *N* times the SMI of one particle, i.e.,

(19) $$\mathrm{SMI}\left(\;N\;independent\;particles\right)=N\times \mathrm{SMI}\left(one\;particle\right)$$

This equation would have been correct if the micro-states of all the particles were independent. In reality, there are always correlations between the micro-states of all the particles: one, due to the *indistinguishability* between the particles, and the second, due to *intermolecular interactions* between the particles. We shall discuss these two sources of correlations separately.

***(i)***  ***Correlation due to indistinguishability***

For a system of *N* simple particles, the indistinguishability of the particles introduces a “mutual information” of the form:

(20) $$I\left(1;2;\ldots ;N\right)=\log N!$$

The SMI for *N* indistinguishable particles is:

(21) $$H\left(N\;particles\right)=\sum_{i=1}^{N}H\left(one\;particle\right)-\log N!$$

For the definition of “mutual information”, see Ben-Naim (2017 [7]). 

Using the Stirling approximation for logN! (note again that we use here the natural logarithm) in the form: logN!≈Nlog N−N, we have the final result for the SMI of *N* indistinguishable, and non-interacting particles in a box of volume *V*, and temperature *T*:

(22) H1,2,…N=Nlog VN2πmkBTh232+52N

This is a remarkable result. By multiplying the SMI of *N* particles in a box of volume *V* at temperature *T* by a constant factor ($k_{B}$, if we use the natural log, or $k_{B}\log_{e}2$ if the log is to the base 2), one obtains the *thermodynamic entropy* of an ideal gas of simple particles. This equation is equivalent to the one derived by Sackur and by Tetrode in 1912 using the Boltzmann *definition* of entropy (see below Equation (24)). Here, we have derived the entropy function, $S\left(T,V,N\right)$, of an ideal gas from the SMI. 

One can convert this expression to the *entropy function* $S\left(E,V,N\right)$, by using the relationship between the total kinetic energy of all particles in the system and the temperature:

(23) $$E=N\frac{mv^{2}}{2}=\frac{3}{2}Nk_{B}T$$

The explicit entropy function of an ideal gas is obtained from (22) and (23):

(24) SE,V,N=NkBlnVNEN32+32kBN53+ln4πm3h2

This is known as the Sackur and by Tetrode equation. It is the fundamental equation of an ideal gas in an isolated system. One can use this equation as a *definition* of the entropy of an ideal gas of simple particles characterized by constant energy, volume, and the number of particles. Note that when we combine all the terms under the logarithm sign, we should obtain a dimensionless quantity. For more details, see Ben-Naim (2017) [7]. It should be noted that this entropy function is a monotonically increasing function of *E*, *V,* and *N*, and the curvature is negative with respect to each of these variables. This is an important property of the entropy function which we will not discuss here. 

The next step in defining entropy, based on SMI, is to add to the entropy of an ideal gas the *mutual information* due to intermolecular interactions. We shall not discuss it here. For details, see Ben-Naim (2017) [7].

We can summarize the ABN *definition* of entropy as follows. We start with the probability distribution of the locations and momenta of all the particles. With this distribution, we define the SMI, take the distribution that maximizes the SMI, and add two corrections to obtain the entropy of an ideal gas at *equilibrium*. One can easily extend this definition to a system of interacting particles by adding the appropriate mutual information. 

This *new definition* of entropy is also equivalent to Clausius’ and Boltzmann’s definitions in the sense that results calculated from the new definition conform with the results calculated with the older definitions.

It should be noted that in the derivation of the entropy, we used the SMI twice: first, in calculating the distribution that maximizes the SMI, then in evaluating the maximum SMI corresponding to this distribution. The distinction between the concepts of SMI and entropy is absolutely essential. Referring to the SMI (as many do) as entropy inevitably leads to such an awkward statement: the maximum value of the entropy (meaning the SMI) is the entropy (meaning, the thermodynamic entropy). The correct statement is that the SMI associated with locations and momenta is defined for any system, small or large at equilibrium, or far from equilibrium. This SMI, not the entropy, evolves into a maximum value when the system reaches equilibrium. At equilibrium, the SMI becomes proportional to the entropy of the system. The entropy obtained in this procedure is referred to as the Shannon-based or the ABN definition of entropy.

Another example of an awkward situation occurs when one calls the SMI, “entropy”. In such a case, one will refer to ln6 as the entropy of a fair die. This is clearly very different from the thermodynamic entropy of the same die. Now consider the following process. If we increase the temperature of the die, does the entropy of the die change? One cannot answer this question without making a distinction between the SMI associated with the distribution of the six outcomes on the faces of the die, and the entropy which is defined by the distribution of locations and momenta of all the particles in the die. The entropy of the die will change in this process, but the SMI, which is ln 6, remains unchanged.

Since entropy is up to a constant, a special case of an SMI, it follows that whatever interpretation one accepts for the SMI will automatically be applied to the concept of entropy. The most important conclusion of this definition is that entropy, being a state function, *is not a function of time*. Entropy does not change with time, and entropy does not have a tendency to increase. It is very common to say that entropy increases towards its maximum at equilibrium. This is wrong. The correct statement is: The entropy is proportional to *the maximum SMI!* As such, it is not a function of time.

Since we used the distribution that maximizes the SMI in the process of defining the entropy, and since this distribution is the same as the distribution at equilibrium, it follows that entropy is defined for a well-defined thermodynamic system at equilibrium. It is also clear that entropy is not defined for any living system, and not for the entire universe. Unfortunately, many recent popular science books invoke the concept of entropy to “explain” many unexplainable phenomena associated with life. Needless to say, this has contributed in making entropy the most mysterious concept in science. 

**Figure 3:**
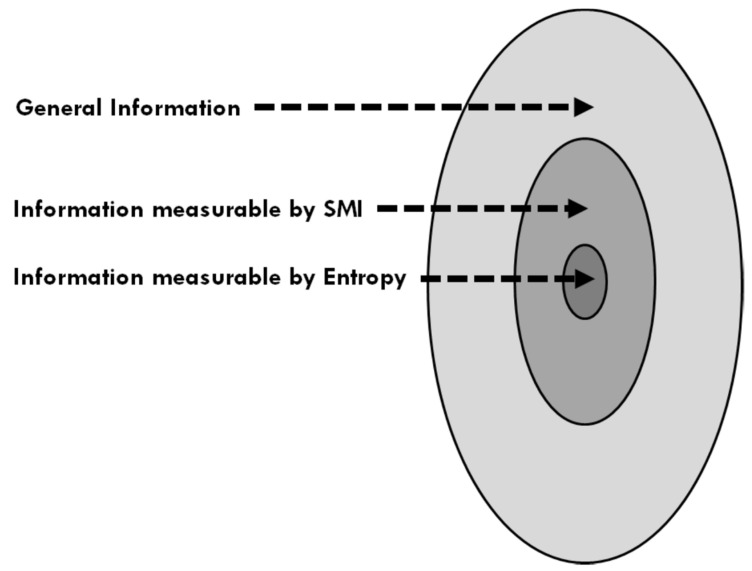
Schematic description of the “relative sizes” of the general information, the information that is measurable by SMI, and the information measurable by entropy.

We interpreted the SMI as a measure of information associated with a probability distribution in Section 2. Similarly, the entropy may be interpreted as a measure of information associated with specific distributions (e.g., of locations and momenta of particles in an ideal gas of simple particles). Figure 3 shows a schematic relation between the “sizes” of three kinds of information.

## 4. The Application of Entropy and the Second Law to Life 

In this section, we discuss the misapplication of SMI, entropy, and the Second Law for entire living systems. As we have seen in Section 2 and Section 3, the concept of SMI may be applied to any system for which a well-defined probability distribution is defined. The entropy, in any one of its definitions, may be applied to systems that are well defined thermodynamically. This follows from the fundamental property of entropy as a *state function*. This means that once we know the state (meaning the macro-state) of a system, its entropy is already determined. We might not be able to calculate the entropy of such a system, but we always assume that it is defined in principle.

The question posed in this section is very simple. Is an entire living system a well-defined thermodynamic system? To the best of my knowledge, no one has ever either measured or calculated the entropy of the entire living system, not a fly, not a dog, and certainly not a human being. The reason is very simple. There are many aspects of living systems, including the characterization in terms of thermodynamic parameters we know nothing of. 

Admittedly, we have gained a huge amount of *information* on many biological systems. We can also apply the concept of SMI to some specific aspects of a living system, e.g., when we know the distribution of some chemicals in the body of an animal, or when we know the probability distribution of letters in the DNA. In such a case, it is meaningful to apply the corresponding SMI, as indeed was done (see below).

Additionally, one can calculate entropy changes for some well-defined processes such as chemical reactions when studied in vitro, i.e., in an isolated and well-controlled system in the laboratory. However, we do not know how to study processes such as *thinking* or *feeling* in a well-controlled system in the laboratory. The reason is that even if we knew exactly the temperature, pressure, and chemical composition of all the components in our body, we would fall short of characterizing the thermodynamic macro-state of the entire living system.

Thus, although it is clear to anyone who has studied thermodynamics that an entire living system is *not* within the “framework of applicability” of entropy and the Second Law, many authors do write about the entropy changes associated with our thoughts, feelings, etc. The most extreme examples may be found in Atkins’ writings:

Atkins (1984) [15], in the introduction to his book, writes:

“*In Chapter 8 we also see how the Second Law accounts for the emergence of the intricately ordered forms characteristic of life*.”

Obviously, this is an unfulfilled promise. The Second Law does not account for the emergence of “ordered forms characteristic of life.” 

Furthermore, in a more recent book, Atkins (2007) [16] writes:

“*The Second Law is of central importance…because it provides a foundation for understanding why **any** change occurs…the acts of literary, artistic, and musical creativity that enhance our culture*.”

Finally, in both of the abovementioned books [15,16], Atkins writes about the Second Law:

“*…no other scientific law has contributed more to the liberation of the human spirit than the Second Law of thermodynamics*.”

All these quotations are extremely impressive, yet are totally empty statements. The Second Law *does not* provide an *explanation* for the “formulation of a thought”, and certainly not the “acts of literacy, artistic and musical creativity”.

In my opinion, such unfounded claims not only do not make any sense but can discourage people from even trying to understand the Second Law of thermodynamics. Everyone knows that life phenomena involve extremely complicated processes; many aspects of life, such as thoughts and feelings, are far from being understood. Therefore, the undeliverable promise of explaining life by the Second Law will necessarily frustrate the reader to the point of concluding that entropy and the Second Law, as life itself, are impossible to understand.

In the rest of this section, I will quote a few more statements which at best can be said to be misapplications of entropy and the Second Law to living systems. A more detailed review of such misapplications may be found in references [1,5]. 

Crick (1981) [17] wrote a short and illuminating book on “Life itself”, where he discussed in plain language the origin of life, the nature of life, and the definition of life. 

Regarding a “definition” of life, Crick writes:

“*It is not easy to give a compact definition of either ‘life’ or ‘living’*.”

The “atom” of all living systems is the *cell*. At the heart of a cell is the nucleus, and at the heart of the nucleus resides the so-called “book of life”, a sequence of letters that contains the “*information”* on that particular organism. This information is written in a four-letter language: A, T, C, and G. Each of these letters is a different molecule (base), and the entire “book” is the DNA molecule. The DNA of human beings contains billions of letters or bases.

The DNA is perhaps the molecule most associated with *information*, both in its colloquial sense, as well as in the theoretical informational sense. One can also assign *entropy* to a segment of, or to the entire DNA. However, this entropy has *nothing* to do with the “information” content of the DNA.

Perhaps the first application of information theory to the DNA was carried out by Gatlin (1972) [18], who wrote a book on “Information Theory and the Living System”. The opening sentence of the book is:

“*Life may be defined operationally as an information processing system–a structural hierarchy of functional units–that has acquired through evolution the ability to store and process the information necessary for its own accurate reproduction. The key word in the definition is information*.”

Although it is certainly true that living organisms process information, this is not the *only* characteristic function of the organism. In general, one cannot use a *characteristic* property of a living system as a *definition* of a living system.

Gatlin treats the sequence of bases in the DNA in the same manner as any sequence of symbols, or letters in any language. In the last part of the introduction, the author introduces the concept of entropy as used in information theory.

“*The unifying thread of our story is the entropy concept. As Homo sapiens, we have always believed that we are higher organisms. After all, we are more complex, more differentiated, more highly ordered than lower organisms. As thermodynamicists, we recognize these words and realize that the concept of entropy must somehow enter into our explanation. We have always had the vague notion that, as higher organisms have evolved, their entropy has in some way declined because of this higher degree of organization. For example, Schrödinger made his famous comment that the living organism ‘feeds on negative entropy’*.”

In my view, Gatlin confuses the thermodynamics entropy with the informational entropy (the SMI). Furthermore, the author expresses the common misconception about “higher organism”, “higher degree of organization” and “lower entropy”. 

When discussing the question “What is life?”, one cannot avoid mentioning the most famous and influential book written by Schrödinger (1943) [19].

This book is based on lectures delivered by Schrödinger in Dublin in 1943. This book was most influential for a long time and probably laid the cornerstone for the creation of the whole field of molecular biology. It has also encouraged many physicists to apply the methods of physics to biology. In this section, I will present some of the highlights of Schrödinger’s book. More details may be found in Ben-Naim (2020) [1,5].

In Chapter 6 of his book titled “Order, disorder and entropy”, Schrödinger starts with the very common misinterpretation of the Second Law in terms of the tendency of a system to go from *order to disorder*:

“*It has been explained in Chapter 1 that the laws of physics, as we know them, are statistical laws. They have a lot to do with the natural tendency of things to go over into disorder*.” “*Life seems to be orderly and lawful behavior of matter, not based exclusively on its tendency to go over from order to disorder, but based partly on existing order that is kept up*.”

The idea that life withstands the “natural tendency to go from order to disorder” appears very frequently in the literature. Unfortunately, there exists no law of nature that dictates going from “order to disorder” in the first place. The tendency of entropy to increase (in some processes) applies to an *isolated system*, certainly not to a living system which is an open system and far from equilibrium. 

On page 74, Schrödinger explicitly relates the Second Law with the behavior of living systems:

“*The general principle involved is the famous Second Law of Thermodynamics (entropy principle) and its equally famous statistical foundation*.” “*It is avoiding the rapid decay into the inert state of ‘equilibrium’ that an organism appears to be enigmatic; so much so, that from the earliest times of human thought some special non-physical or supernatural force (vis viva, entelechy) was claimed to be operative in the organism, and in some quarters is still claimed*.”

Then, Schrödinger asks:

“*How does the living organism avoid decay?”*

On page 76, Schrödinger answers:

“*What then is that precious something contained in our food which keeps us from death? That is easily answered. Every process, event, happening–call it what you will; in a word, everything that is going on in Nature means an increase of the entropy of the part of the world where it is going on. Thus, a living organism continually increases its entropy–or, as you may say, produces positive entropy–and thus tends to approach the dangerous state of maximum entropy, which is death. It can only keep aloof from it, i.e., alive, by continually drawing from its environment negative entropy–which is something very positive as we shall immediately see. What an organism feeds upon is **negative entropy**. Or, to put it less paradoxically, the essential thing in metabolism is that organism succeeds in freeing itself from all the entropy it cannot help producing while alive*.”

In my view, Schrödinger’s answer was a dismal failure to the question he posed. In the first place, I certainly do not agree that everything that goes on in Nature means an “increase of the entropy”. Second, I do not agree that living things “produce positive entropy”. Third, I have no idea what “the dangerous state of maximum entropy” (for a living organism) means. Finally, I do not agree that the only way it can stay alive is by drawing *negative entropy* from its environment. Unfortunately, such statements are all meaningless. Entropy, by definition, is a positive quantity. There is no *negative entropy*, as there is no negative mass or negative volume. Of course, there are processes involving negative *changes* in entropy, but there exists no *negative entropy*!

Most people praised the book, although some expressed their doubts about its content. Perhaps the most famous skeptic of Schrödinger’s contribution (to the understanding of life, of course) was Pauling. In a biography of Linus Pauling, Hager [20] mentioned Pauling’s view of Schrödinger’s book.

“*Pauling thought the book was hogwash. No one had ever demonstrated the existence of anything like “negative entropy”, … Schrödinger’s discussion of thermodynamics is vague and superficial… Schrödinger made no contribution to our understanding of life*.”

I fully agree with Pauling’s views about Schrödinger’s usage of thermodynamics in connection with life.

To conclude this discussion of “What is Life”, let me say that entropy, which is a *meaningful quantity,* becomes *meaningless* when applied to a living system. It is a fortiori true when a *meaningless quantity* such as *negative entropy* is applied to a living system. 

Let me add my view regarding the association of entropy and the Second Law with the question of Life. I believe that if ever a complete physical theory of life shall be developed it will heavily rely on information theory, and not on entropy and the Second Law of thermodynamics. 

Brillouin (1962) [21] suggested using *neg-entropy* instead of “negative entropy” which is supposed to be interpreted as information. Brillouin goes even further and claims that:

“*If a living organism needs food, it is only for the negentropy it can get from it, and which is needed to make up for the losses due to mechanical work done, or simple degradation processes in living systems. Energy contained in food does not really matter: Since energy is conserved and never gets lost, but negentropy is the important factor*.”

While I am still baffled with the concept of *negative entropy*, or the shorter version of *negentropy*, I was greatly relieved to read the explanation (Hoffmann, 2012) [22]:

“*Life uses a low-entropy source of energy (food or sunlight) and locally decreases entropy (created order by growing) at the cost of creating a lot of high-entropy waste energy (heat and chemical waste)*.”

Thus, in modern books, the meaningless notion of *negative entropy* (or neg-entropy) is replaced by the more meaningful term of *low entropy*.

Does a living organism feed on *low entropy* food? If you are convinced that feeding on low entropy food is the thing that keeps you alive, your soup should be (as well as your coffee and tea) as cold as possible. This will ensure you are feeding on food with the lowest entropy possible. 

The whole idea of feeding on either the meaningless “negative entropy” or the meaningful “low-entropy” food is based on the misinterpretation of entropy as a measure of disorder or disorganization. Qualitatively speaking, we feel that a living system has some degree of *order* and *organization*. This is not the same as saying that a living system has *lower entropy*. 

Seife (2007) [10] dedicates a whole chapter in his book to “Life”. Before he discusses life itself, he states that “*thermodynamics is, in truth a special case of information theory*”. I do not agree with this statement. As we noted above, entropy may be viewed as a special case of SMI. Clearly, entropy is one concept within thermodynamics. While the SMI is also one concept in information theory, it does not follow that thermodynamics is a special case of information theory. In my opinion, Seife repeatedly confuses the concepts of information, SMI, and entropy in the entire book. 

Finally, I feel it is my obligation to science to comment on a recent book titled “*Genetic Entropy and the Mystery of the Genome*” by Sanford (2005) [23]. This book uses the term Entropy only for the purpose of *embellishing* the book’s title. The book itself is a flagrant misuse, or perhaps abuse of the term entropy. For details, see reference [5].

## 5. The Universe 

Before we discuss the application of entropy and the Second Law to the entire universe, it is appropriate to go back to Clausius (1865) [24], who coined the term entropy and is credited for his famous statement about the Second Law:

**“*The entropy of the Universe always increases*.”**

Recall that Clausius did not define entropy, but a small change in entropy for a particular process (see Section 3). It is clear that Clausius did not (and in fact, could not) understand the meaning of the concept that he coined on a molecular level. Before the Second Law was finally formulated in terms of entropy, it was known that for many spontaneous processes occurring in isolated systems, the entropy increases. From these facts, one can generalize that in any spontaneous process in an isolated system the entropy increases. See Callen [12] and Ben-Naim [3]. Clearly, the stipulation that the system is isolated is essential. If the system is not isolated, the entropy can either go up or down, and the so-called entropy formulation of the Second Law would not apply.

In Section 4, we emphasized that the entropy may not be applied to an *entire* living system, not because it is not an isolated system (hence, the entropy formulation of the Second Law does not apply) but rather because a living system is not a well-defined thermodynamic system. Therefore, entropy as a state function is not applicable.

Once we accept that entropy is a state function, it becomes clear that we cannot apply it to the *entire* universe. As we commented in Section 4, one can talk about the SMI as well as the entropy of specific *parts* of the universe, but we cannot apply these concepts to the *entire* universe. Why? Simply because we do not know how to characterize the entire universe in terms of thermodynamic parameters. In fact, we do not know whether the universe is finite or infinite. Therefore, it is meaningless to talk about the volume or the energy of the entire universe. The same is true for entropy; the entire universe is outside the “*framework of applicability*” of the concept of entropy.

Ridiculous as it may sound, some authors not only discuss the entropy of the universe, but also provide numbers of the entropy of the universe in the past, present, and even at, or near the Big Bang.

Everyone knows that the universe is unimaginably vast. We know a great deal about the universe, more than can fit in one voluminous book. Yet, as in the case of life, as discussed in the previous section, there are many fundamental questions about the universe, the answers to which are unknown, and perhaps will never be known. Is the universe finite or infinite? Can we predict the future of the universe? Can we ever know the exact “beginning” of the universe? Is the total energy and mass in the universe finite? Additionally, perhaps the least talked about question is: Are the laws of physics as we know them today the same everywhere in the universe? Will these laws be the same in the distant future, and were they the same in the distant past? 

All these unknowns are more than enough to preclude the application of both the SMI and entropy to the entire universe. Yet, authors of popular science books allow themselves to write whatever comes to their mind on the *entropy* and SMI of the entire universe. 

Let us mention a few typical statements.

Lloyd’s (2006) [8] book “Programming the Universe” contains these opening sentences:

“*This book is the story of the universe and the bit. The universe is the biggest thing there is, and the bit is the smallest possible chunk of information*.”

Indeed, the universe is, almost by definition, the “biggest thing”, but the “bit” is *not* the smallest possible chunk of information, as much as the centimeter is not the smallest chunk of length, or the second the shortest chunk of time. Then, the author continues:

“*The universe is made of bits. Every atom and elementary particle registers bits of information*.” “*The universe is a quantum computer.” This begs the question: “What does the universe compute? It computes itself. The universe computes its own behavior. As soon as the universe began, it began computing…*”

I do not agree with such statements. They all sound impressive and are frequently quoted by many authors. I have no idea how the universe “computes”, and assuming that it does, I have no idea what it means to compute itself.

Atkins (2007) [16] dedicates a whole book to the “Four Laws that Drive the Universe”. In the book’s preface, the author states:

“*The Second Law is one of the all-time great laws of science, for it illuminates why anything–anything from the cooling of hot matter to the formulation of a thought–happens at all*.”

Additionally, on page 62, the author writes:

“*The entropy of the universe increases in the course of any spontaneous change. The key word here is **universe**; it means, as always in thermodynamics, the system together with its surroundings. There is no prohibition of the system or the surroundings **individually** undergoing a decrease in entropy provided that there is a compensating change elsewhere*.”

These are wild and meaningless statements. The “*key word here is universe*”. In my opinion, this “keyword” invalidates the quoted statement of the Second Law. We know that entropy increases in a spontaneous process in an *isolated* system. In this formulation of the Second Law, the “keyword”—“universe”—does not appear, and therefore is superfluous.

There are actually two issues on which most writers on the entropy of the universe went astray. One is the *value* of the entropy of the universe, and in which direction it is expected to change in millions or billions of years from now. The second is the *fate* of the universe. What will happen? Is the universe doomed to reach its “thermal death?” 

Regarding the issue of the entropy of the universe, all the conclusions reached in the previous section regarding the *entropy* of *life* are a fortiori true for the entropy of the universe. Why?

First, because life is *included* in the universe. Since it is meaningless to talk about the entropy of all living organisms in the universe, it is also meaningless to talk about the entropy of the entire universe. Second, in addition to having life within the universe, we still do not know whether the universe is finite or infinite. We certainly know that the universe is not in an equilibrium state. In addition, we have no idea whether the universe will ever reach an equilibrium state.

Since we do not know whether the universe will ever reach an equilibrium state, authors should refrain from frightening people about the universe reaching a *thermal death*, or that the *ravaging* power of entropy is a harbinger of the end of life and everything that life has created in our planet or in the entire universe. 

Take note that I do not claim that the universe might not “die” (whatever this might mean) some billions of years from now. This might happen. I object to the prediction of the death of the universe *based* on the Second Law of Thermodynamics, and that this fate is inevitable. If thermal death will happen, it will not happen because of the “ravages of the entropy, or because of the Second Law.

The phrase “ravages of entropy” has become commonplace in many recent popular science books. People who talk about the ravages of entropy also talk about the ravages of time (this becomes almost the same if one identifies entropy or the Second Law with the arrow of time).

Colloquially, the expression “ravages of time” is a very common figure of speech used by scientists, as well as by non-scientists. There is nothing wrong with this for as long as one means that some processes seem to lead to deterioration, decay, or death. However, there is no such general rule or a law of nature that states that things are ravaged by time. One can equally say that many phenomena such as flourishing, blooming and births are the “creation of time.” Thus, for some processes, one can say figuratively that they are the result of the “ravages of time”, but for others, one can also say that they are the results of the “creative power of time”—again, only figuratively. Time, by itself, does not ravage anything, nor does it create anything.

Entropy, on the other hand, is far more “innocent” than time. It does not ravage anything, and it does not create anything! It does not create “order out of disorder”, nor “disorder out of order”. In short, entropy does not *do* anything, not even figuratively, as much as the *length* of this article, the number of pages, or the number of letters, do not ravage anything, nor create anything. Yet, you read in many popular science books about the “ravages of entropy”, giving the impression that the almighty entropy has the power to destroy anything while it grows (see Epilogue in Ben-Naim (2020) [5]). Such statements result from two fundamental misconceptions of the Second Law, a tendency toward disorder, and the association of entropy with the arrow of time.

All that I have said above about the distant *future* of the universe also applies to the distant *past* of the universe. People deduce from the fact that the entropy of the universe always increases, that the value of the entropy must have been very low in the early times of the universe. This is known as the Past Hypothesis [25]. This idea has filled the entire book by Carroll 2010) [26]. Briefly, the Past Hypothesis, according to Carroll, states:

“*When it comes to the past, however, we have at our disposal both our knowledge of the current macroscopic state of the universe, plus the fact that the early universe began in a low-entropy state. That one extra bit of information, known simply as the “Past Hypothesis”, gives us enormous leverage when it comes to reconstructing the past from the present*.”

Do we have the “current macroscopic state of the universe?” I doubt the veracity of this statement. The “fact” that the early universe began in a low entropy state is not a fact! At best, this is a wild hypothesis, probably even a meaningless one. Even if we had known the exact macroscopic state of the universe, I doubt that the Past Hypothesis could help in reconstructing the past from the present. On the same page, we find another “punch line”:

“*The punch line is that our notion of free will, the ability to change the future by making choices in a way that is not available to us as far as the past is concerned, is only possible because the past has a low entropy and the future has a high entropy. The future seems open to us, while the past seems closed, even though the laws of physics treat them on an equal footing*.”

The truth of the matter is that we have no idea about the entropy of the past, nor about the entropy of the future. Even if we knew these, I doubt that these entropies would have anything to do with “free will?”

Here, are more quotations from Carroll’s book (2010) [26]:

“*If everything in the universe evolves toward increasing disorder, it must have started out in an exquisitely ordered arrangement. This whole chain of logic, purporting to explain why you can’t turn an omelet into an egg, apparently rests on a deep entropy, very high order.*

*The arrow of time connects the early universe to something we experience literally every moment of our lives…. The arrow of time is the reason why time seems to flow around us, or why (if you prefer) we seem to move through time. It’s why we remember the past, but not the future. It’s why we evolve and metabolize and eventually die. It’s why we believe in cause and effect, and is crucial to our notions of free will*.”

The fact is that in a controlled experiment carried out in a laboratory in an isolated system, the entropy increases. From these experiments, we cannot conclude that the universe started out in an “exquisitely ordered arrangement”, and besides, an untended room does not get messier over time! These statements involve typical misinterpretations of entropy in terms of disorder and applying the concept of entropy (or disorder) to where it does not apply. 

In contrast to the idea of the low-entropy past, we find in Greene (2004), page 100, the following:

“*Thus, not only is there an overwhelming probability that the entropy of a physical system will be higher in what we call the future, but there is the same overwhelming probability that it was higher in what we call the past*.”

In my opinion, such statements create the impression that somehow the entropy of a system is a well-known function of time (as it is actually illustrated in Greene’s book, Figure 6.2). This is not so. When a such argument is applied to the *whole universe*, it turns from being wrong to meaningless. Since the entropy of the universe is not defined, we cannot say anything about its value either in the past or in the future. 

Personally, I do not subscribe to either the “Past Hypothesis”, or to the “past chaos” views. Regarding the entropy, I can say more categorically that not only do we not know the *value* of the entropy, but the entropy of the universe is simply undefined.

Carroll (2010) [26], in discussing the Past Hypothesis, makes the analogy between the *expansion* of the *universe* and the *increase* in *entropy*. This analogy is invalid for the following reason. We observe the expansion of the Universe. We can extrapolate back in time and conclude that some 14–15 billion years ago, the whole universe was highly condensed. This extrapolation is valid provided that the universe always expanded, and that the laws of physics, as we know them today, were the same billions of years ago. With these assumptions, we may speculate about the Big Bang theory.

Recently, some physicists have promoted the ideas about the multiverses, or the parallel universes which is essentially an alternative to the Copenhagen interpretation of quantum mechanics. Of course, no one knows anything about these parallel universes, or whether or not they are similar to “our universe”, or perhaps completely different universes with different sets of laws of physics, chemistry, or biology. Needless to say, we cannot say anything about the entropy of these universes.

Having dismissed the entropy of the universe, there still remains the question of the applicability of the SMI to the universe. There is no problem in applying the SMI to any well-known probability distribution. It could be the SMI based on the distribution of locations and velocities of particles, or the distribution of energies in any part of the universe. However, the reference to the SMI of the entire universe is meaningless *unless we specify the relevant distribution*. Unfortunately, the literature is replete with statements about the entropy of the universe, the information of the universe, and the SMI of the universe. For examples and more details, see Ben-Naim [1,5]. 

## 6. Summary

“*The entropy of the universe always increases*.”

This statement has been quoted innumerable times. Unfortunately, there is no basis to either justify it or to lend any meaning to it. The universe is not a well-defined thermodynamic system. It is not clear whether the universe is finite or infinite, nor whether or not it will ever reach an equilibrium state. Therefore, it is meaningless to speculate about the changes in the entropy of the universe.

Clearly, the thermodynamic entropy of the universe cannot be measured. We cannot conduct experiments on the entire universe. The same is true for the Boltzmann entropy. Can we calculate the number of states of the universe?

My general conclusion in this section is the same as in Section 4. We do not know how to define the thermodynamic *state* of being alive. Therefore, we cannot talk about the entropy of a living organism. This conclusion is a fortiori true for the entire universe.

In response to some reviewers’ comments, I am adding the following two comments:

1. The derivation of entropy from the SMI was first published by Ben-Naim (2008). It is not the same as the so-called Gibbs entropy. Gibbs’ entropy was derived from Boltzmann’s definition of entropy and is only valid for equilibrium states. Gibbs’ entropy uses different equilibrium distributions of *macroscopic* parameters such as total energy, volume, and the total number of particles. The ABN definition of entropy is based on the SMI defined on the distribution of locations and momenta of the particles. It does not use the Boltzmann entropy, and therefore it is different from both the Boltzmann and the Gibbs entropy.

2. All that was said about the independence of entropy on time, and about the inapplicability of entropy to life phenomenon, and for the entire universe is valid for any definition of entropy. It is based on the fact that entropy is a *state function,* as recognized by all those who are familiar with thermodynamics. Being a *state function,* is a property of any entropy, not only of the ABN’s entropy, as one reviewer commented.

## Data Availability

Not applicable.
